# Thermoregulation and associated disorders: 3PM-guided holistic approach bridging innovative and traditional Chinese medicine

**DOI:** 10.1007/s13167-026-00453-8

**Published:** 2026-04-15

**Authors:** Zhuo Wang, Hongtao Liu, Yueqiang Xu, Weijie Cao, Youxin Wang, Haifeng Hou, Xiuhua Guo, Olga Golubnitschaja, Wei Wang, Yuguang Du, Suboptimal Health Study Consortium, European Association for Predictive, Preventive, Personalised Medicine

**Affiliations:** 1 Glyco-Bioengineering Group, Institute of Process Engineering, Chinese Academy of Sciences, Beijing, China; 2 Li Shizhen Institute, Hubei University of Chinese Medicine, Wuhan, China; 3 Beijing BAHEAL GLYCOMTECH Co., Ltd, Bejing, China; 4 School of Public Health, North China University of Science and Technology, Tangshan, China; 5 School of Public Health, Shandong First Medical University & Shandong Academy of Medical Sciences, Tai’an, China; 6 Predictive, Preventive and Personalised (3P) Medicine, Department of Radiation Oncology, University Hospital Bonn, Rheinische Friedrich-Wilhelms-Universität Bonn, Bonn, Germany; 7 Institute for Glycome Study, The First Affiliated Hospital, Shantou University Medical College, Shantou, China; 8 Chemistry and Chemical Engineering Guangdong Laboratory, Shantou, Guangdong China; 9 Nutrition & Health Innovation Research Institute & School of Medical and Health Sciences, Edith Cowan University, Joondalup, Australia

**Keywords:** Predictive Preventive Personalized Medicine (PPPM / 3PM), Paradigm shift from reactive to proactive healthcare, Traditional Chinese Medicine, *Yin–Yang*, Cold–Heat syndromes, TRP channels, Ion channels, Epigenetics, Glycobiology, Systemic effects, Holistic approach, Individualized patient profile, Patient phenotyping and stratification, Improved individual outcomes

## Abstract

Accurately performed thermoregulation is life-important for the human body. Therefore, a relatively narrow temperature range of 36.5–37 °C, which all our biochemical reactions are adapted to, is rigorously kept by the body allowing for the most effective kinetics of all physiological processes. In contrast, feeling inappropriately cold or too hot in the environment with comfortable temperature ranges are symptoms of an altered or even disordered thermoregulation described for a number of syndromes as well as patient cohorts. The rationale of the paper is to contribute to the paradigm shift from reactive to proactive healthcare considering thermoregulation deficits as an important diagnostic and prognostic indicator to be explored and utilized for patient phenotyping and stratification followed by tailored treatment algorithms in primary and secondary care. The conceptual framework of *Yin–Yang*/Cold–Heat syndromes in Traditional Chinese Medicine (TCM) provides a holistic description of physiological balance, adaptability, and pathological deviation. Recent advances in molecular physiology and biotechnology now permit these ancient classifications to be reframed as quantifiable, systems-level biological states. This review integrates thermosensitive transient receptor potential (TRP) channels with ion-channel networks, inflammatory signaling, and emerging multiomics regulation to reinterpret Cold–Heat syndromes through a modern biotechnological lens. We further incorporate the paracentral dogma concept—highlighting epigenetic, proteomic, and glycomic regulation beyond the classical DNA–RNA–protein axis—to explain how non-template-driven molecular layers dynamically tune TRP channel sensitivity and downstream inflammatory balance. Drawing on advances in genomics, proteomics, glycomedicine, and systems engineering, we propose that Cold–Heat states represent stable yet reversible molecular attractors shaped by environmental exposure, metabolic state, and post-translational modification. Finally, we outline translational opportunities including TRP-based biosensors, epigenetic, protein and glycan biomarkers, and AI-driven Cold–Heat stratification platforms. This integrative framework positions TCM-inspired pattern differentiation as a scalable systems biology paradigm with direct relevance to predictive, preventive and personalized healthcare.

**Graphical Abstract:**

A left–right Cold (Yin) to Heat (Yang) continuum integrates layered regulation from genetics, epigenetics, and glycobiology through TRP/ion channels to cytokine-driven clinical phenotypes, visually unifying TCM theory with modern molecular biotechnology.

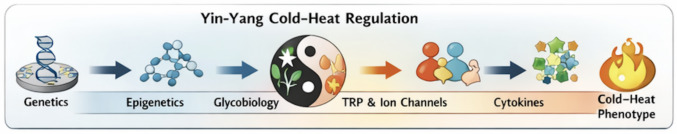

## Preamble

Accurately performed thermoregulation is life-important for the human body. Therefore, a relatively narrow temperature range of 36.5–37 °C, which all our biochemical reactions are adapted to, is rigorously kept by the body allowing for the most effective kinetics of all physiological processes. In contrast, feeling inappropriately cold or too hot in the environment with comfortable temperature ranges are symptoms of an altered or even disordered thermoregulation described for a number of syndromes as well as for several patient cohorts. Accumulated studies demonstrate that, for example, breast cancer (BC) patients are frequently deficient in achieving thermal comfort: whereas disease-free attenders are well comfortable with ambient temperature conditions, BC patients feel either excessively hot or cold [1, 2]. Specifically in case of female health, the hot feeling is relatively well studied in context of menopausal symptoms (hot flashes). In contrast, feeling inappropriately cold is significantly less well explored [1]. These deficits have been addressed by the international multi-professional EPMA project dedicated to the association between individualized BC patient profiles and the Flammer syndrome phenotype (FSP) investigating systemic effects and implicating 3PM-giuded holistic approach [1, 3–6]. FSP carriers are characterized by disturbed microcirculation as well as significantly increased stress sensitivity, which is functionally linked to mitochondrial stress, mitochondrial impairments and burnout – all highly relevant for a diminished thermoregulation and predisposition to a spectrum of severe pathologies [1, 7]. Contextually, in order to shift the paradigm from reactive to proactive healthcare, mitochondrial biosensorics is considered pivotal for health status monitoring and 3PM-guided approach [8]. On the other hand, significant deficits in thermoregulation are considered an important diagnostic and prognostic indicator to be explored and utilized for patient phenotyping and stratification followed by tailored treatment algorithms in primary and secondary care.

## Introduction

**Fig. 1:**
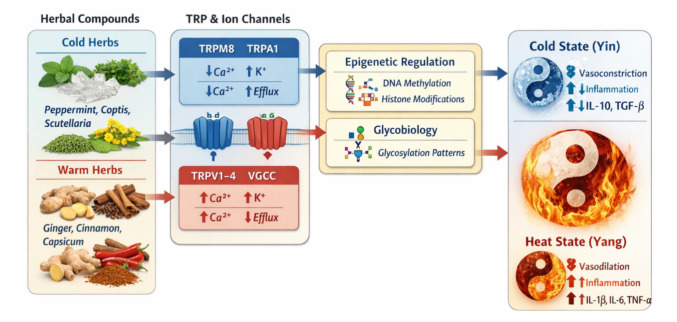
Mapping of TCM Cold–Heat syndromes to thermosensitive TRP channels. Cold (*Yin*) states are associated with TRPM8/TRPA1 activation, K⁺ efflux, vasoconstriction, reduced metabolism, and anti-inflammatory cytokines. Heat (*Yang*) states correspond to TRPV1–3 activation, Ca^2^⁺ influx, vasodilation, hypermetabolism, and pro-inflammatory cytokine release. This schematic illustrates how traditional Chinese medicinal (TCM) herbs influence systemic Cold/*Yin* and Heat/*Yang* phenotypes through multi-layered molecular mechanisms. **Left panel:** Representative Cold (blue) herbs (e.g., peppermint, Coptis, Scutellaria) and Warm (red) herbs (e.g., ginger, cinnamon, capsicum) are classified by their traditional temperature nature. **Middle panel:** Herbal bioactive compounds modulate **thermosensitive TRP channels** (TRPV1–4 for Heat/*Yang*; TRPM8, TRPA1 for Cold/*Yin*) and downstream **ion fluxes** (Ca^2^⁺ influx, K⁺ efflux), altering neuronal excitability, vascular tone, and metabolic activity. TRP/ion signaling is further regulated by **epigenetic mechanisms** (DNA methylation, histone modifications) and **glycobiological modifications**

Cold and Heat syndromes represent fundamental pattern categories in Traditional Chinese Medicine (TCM), embedded within *Yin–Yang* theory and used to guide individualized therapy [9, 10]. These patterns integrate sensory perception, circulation, metabolism, and immune tone, emphasizing dynamic regulation and organism–environment interaction. In modern biomedicine, thermosensitive transient receptor potential (TRP) channels (TRPV1–4, TRPM8, TRPA1) function as molecular thermosensors and polymodal integrators that regulate neuronal excitability, vascular tone, inflammatory signaling, and metabolic activity [11–13]. Increasing evidence suggests that TCM Cold–Heat patterns can be interpreted as emergent properties of TRP-centered ion channel and inflammatory networks. Such an interpretation aligns with the translational scope of systems biology, biomarker development, and integrative bioengineering from the context of Predictive, Preventive and Personalized Medicine (PPPM) [14, 15]. Figure 1 provides an integrative systems-level framework linking TCM herbal classification, thermosensitive TRP channels, ionic signaling, epigenetic/glycobiological regulation, and emergent Cold–Heat phenotypes.

## Cold–Heat syndromes in TCM: A Yin–Yang systems view

*Yin–Yang and Regulatory Balance: Yin and Yang* represent fundamental, complementary functional axes that orchestrate systemic physiological regulation [15]. *Yin* embodies cooling, inhibitory, nourishing, and structural processes, providing stability, maintenance, and restoration of bodily tissues. By contrast, *Yang* encompasses warming, activating, transformative, and functional processes, driving metabolic activity, energy mobilization, and physiological responsiveness [16]. Table 1 summarizes the core conceptual and physiological distinctions between *Yin* and *Yang* as high-level regulatory axes.

**Table 1:** Yin vs. Yang: conceptual and physiological characteristics

Dimension	*Yin*	*Yang*
Core attribute (TCM)	Cold, inhibitory, nourishing	Heat, activating, transformative
Functional role	Structural, stabilizing	Functional, dynamic
Metabolic state	Low, energy-conserving	High, energy-consuming
Neural excitability	Reduced	Enhanced
Vascular tone	Vasoconstriction	Vasodilation
Inflammatory bias	Anti-inflammatory	Pro-inflammatory
Adaptation mode	Preservation, tolerance	Reactivity, defense

Cold–Heat syndromes emerge when the *Yin–Yang* balance is disrupted. This imbalance may arise from: 1) *Endogenous deficiency or excess* – e.g., impaired *Yang* activity may lead to cold-dominant (*Yin*-excess) syndromes, whereas Yin deficiency may predispose to heat-dominant (*Yang*-excess) states; 2) *Exogenous climatic influences* – prolonged exposure to cold, heat, dampness, or dryness can perturb internal regulatory dynamics, tipping the system toward Cold or Heat patterns; 3) Dysregulated organ networks – organ interactions, particularly among the liver, spleen, and kidney, play a central role. For instance, insufficient kidney *Yang* can impair warming and fluid metabolism, whereas liver *Yin* deficiency may fail to restrain hyperactive *Yang*, leading to Heat manifestations [9, 10].

Importantly, TCM views Cold–Heat syndromes as systemic functional states rather than localized pathologies. They reflect dynamic homeostatic adjustments, where compensatory mechanisms in one organ system influence others, aligning with modern concepts of network physiology [17, 18]. In this perspective, the *Yin–Yang* axis can be considered a high-level integrator of metabolic, neuroendocrine, immune, and vascular regulatory loops, maintaining systemic equilibrium under varying internal and external pressures. Cold (Yin-dominant) and Heat (Yang-dominant) patterns exhibit distinct systemic *clinical* phenotypes*/phenomenology* [16]. The major clinical, metabolic, and immunological correlates of Cold and Heat syndromes are summarized in Table 2.

**Table 2:** Cold vs. heat syndromes: TCM and biomedical correlates

Feature	Cold Syndrome (*Yin*-dominant)	Heat Syndrome (*Yang*-dominant)
Thermal sensation	Cold intolerance	Heat intolerance
Skin and face	Pale, cool	Red, warm
Pulse	Slow, deep	Rapid, forceful
Metabolism	Hypometabolic	Hypermetabolic
Immune tone	Suppressed or tolerant	Activated or excessive
Inflammatory pattern	Chronic, low-grade	Acute, high-grade

*Cold (Yin-dominant) patterns*: *1)* Macroscopic features: cold intolerance, pale or bluish complexion, cold extremities, fatigue, low energy, and slow, deep pulse; 2) Functional correlates: reduced basal metabolic rate, diminished circulation, slowed enzymatic reactions, and hyporeactive immune responses; 3) Molecular correlates: decreased TRP (transient receptor potential) channel excitability, lower ionic flux (e.g., Ca^2^⁺, Na⁺, K⁺), and attenuated pro-inflammatory cytokine activity. These molecular states provide a mechanistic bridge to observed cold phenotypes [19, 20]. In contrast, Heat (*Yang*-dominant) patterns: *1)* Macroscopic features: heat intolerance, fever, flushed complexion, restlessness or irritability, rapid pulse, and hypermetabolic or hyperinflammatory tendencies; 2) Functional correlates: accelerated metabolism, enhanced cardiac output, elevated thermogenesis, and amplified immune surveillance; 3) Molecular correlates: upregulated TRP channel activity, enhanced ion transport, and elevated pro-inflammatory cytokine milieu, reflecting a system primed for heightened responsiveness [21, 22].

By mapping TCM diagnostic patterns onto molecular network states, these observations provide a translational framework connecting traditional clinical phenotyping with modern physiology and systems biology [15]. This approach allows researchers and clinicians to explore how environmental, genetic, and epigenetic factors modulate the *Yin–Yang* balance and contribute to Cold–Heat susceptibility in individuals [23, 24].

## Thermosensitive TRP channels as molecular substrates of cold–heat sensation

Transient receptor potential (TRP) channels serve as molecular thermosensors, transducing environmental and endogenous thermal cues into cellular and systemic responses [19, 22]. An overview of thermosensitive TRP channels, their activation ranges, representative ligands, and associated Cold–Heat biases is presented in Table 3. Their differential activation underlies the physiological and symptomatic distinctions between Cold (*Yin*) and Heat (*Yang*) states.

**Table 3:** Thermosensitive TRP channels and cold–heat associations

TRP Channel	Activation range	Representative ligands	Functional role	Cold–Heat bias
TRPM8	Cool	Menthol	Cold sensing, vasoconstriction	Cold/Yin
TRPA1	Noxious cold, irritants	Allylisothiocynate; Reactive Oxygen Species	Stress and chemical sensing	Cold/Yin
TRPV1	Noxious heat	Capsaicin	Pain, inflammation, fever	Heat/Yang
TRPV3	Warm	Endogenous lipids	Thermoregulation	Heat/Yang
TRPV4	Warm, osmotic	Mechanical stress	Vascular and metabolic control	Heat/Yang

*Heat-Activated TRP Channels:* Heat-sensitive TRP channels include TRPV1, TRPV2, TRPV3, and TRPV4, which respond to warm-to-noxious temperatures (≈30–52 °C) and are modulated by pro-inflammatory mediators such as prostaglandins, bradykinin, ATP, and endovanilloids [11, 13]. Their Functional and physiological roles: 1) Peripheral vasodilation and thermogenesis: TRPV activation in cutaneous and vascular smooth muscle cells promotes local blood flow and heat dissipation, contributing to the flushed complexion and warmth characteristic of *Yang*/Heat patterns; 2) Neurogenic inflammation: Activation triggers release of neuropeptides like substance P and Calcitonin gene-related peptide (CGRP) from sensory neurons, amplifying local inflammation and immune alertness; 3) Central thermoregulatory signaling: TRPV channels in hypothalamic and brainstem nuclei integrate peripheral temperature signals, modulating autonomic output to maintain systemic thermal balance [13, 19]; 4) Metabolic upregulation: TRPV activation increases mitochondrial activity and cellular metabolism in peripheral tissues, consistent with the hypermetabolic features of Heat syndromes.

Heat-activated TRP channels form a mechanistic bridge connecting environmental warmth, inflammation, and *Yang*-dominant physiological states [12, 20]. Dysregulation may contribute to Clinical and translational relevance, e.g., chronic heat-like syndromes, fever, or hyperinflammatory conditions.

*Cold-Activated TRP Channels:* Cold-sensitive TRP channels primarily include **TRPM8** (activated at ≈15–28 °C) and **TRPA1** (responsive to noxious cold < 17 °C), which are also triggered by chemical agonists such as menthol (TRPM8) and allyl isothiocyanate (TRPA1) [11]. Their Functional and physiological roles include: 1) **Vasoconstriction and heat conservation:** Activation of TRPM8/TRPA1 in vascular smooth muscle and sensory neurons promotes peripheral vasoconstriction, reduced heat loss, and maintenance of core body temperature [12]; 2) **Anti-inflammatory signaling:** These channels can dampen pro-inflammatory cytokine release and reduce immune cell recruitment, reflecting the hypoactive, inhibitory characteristics of *Yin*/Cold states [19, 21]; 3) **Neuronal excitability modulation:** Cold-activated TRPs reduce sensory neuron firing in response to non-noxious stimuli, contributing to decreased peripheral sensitivity and fatigue often observed in Cold syndromes [17, 18]; 4) **Integration with central thermoregulation:** TRPM8 and TRPA1 inputs converge on hypothalamic centers, orchestrating adaptive thermogenic responses (e.g., shivering, brown adipose tissue activation) consistent with cold tolerance mechanisms. Cold-activated TRP channels provide a direct molecular substrate for linking environmental temperature and systemic Cold–Heat physiology [23, 24]. Their modulation may inform therapeutic strategies in conditions such as cold intolerance, Raynaud’s phenomenon, or chronic fatigue syndromes.

### Integration of TRP with TCM physiology:

- ***Yang*/Heat phenotype:** High TRPV activity → vasodilation, hypermetabolism, neurogenic inflammation.
- ***Yin*/Cold phenotype:** High TRPM8/TRPA1 activity → vasoconstriction, anti-inflammatory signaling, metabolic conservation.

**Fig. 2:**
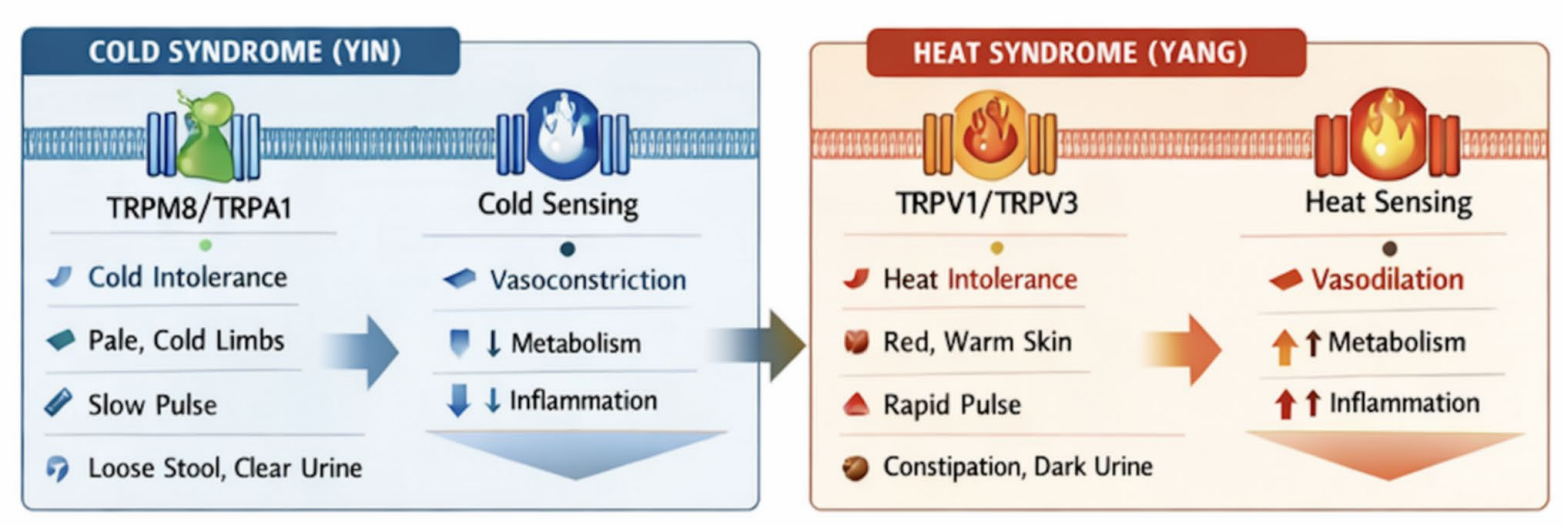
TRP–Ca^2^⁺–K⁺ ion channel network regulating vascular, neuronal, and metabolic outcomes. TRP-mediated Ca^2^⁺ entry amplifies excitatory and inflammatory signaling, while K⁺ channels stabilize membrane potential and suppress excessive activation

By mapping TRP channel activity to *Yin–Yang* states, we, therefore, can frame Cold–Heat syndromes in **molecular terms**, bridging traditional diagnostics with contemporary mechanistic biology (Fig. 2).

## Mechanistic integration: TRP channels, Ca^2^⁺ and K⁺ ion systems

Thermosensitive TRP channels are **non-selective cation channels** with high permeability to **Ca**^**2**^**⁺**, serving as key mediators of temperature-induced cellular responses. Upon activation by heat (TRPV) or cold (TRPM8/TRPA1), TRP channels **depolarize the cell membrane**, which in turn recruits **voltage-gated Ca**^**2**^**⁺ channels (VGCCs)**, amplifying intracellular Ca^2^⁺ signaling [25].

**Downstream effects of Ca**^**2**^**⁺ influx include:** 1) **Neuropeptide release** – Ca^2^⁺-dependent exocytosis of substance P, CGRP, and other mediators promotes vasodilation, inflammation, and neurogenic signaling. 2) **Activation of Ca**^**2**^**⁺-dependent kinases** – including CaMKII and PKC, which modulate ion channel activity, transcription factors, and metabolic enzymes [26, 27]; 2) **Transcriptional programs** – elevated intracellular Ca^2^⁺ triggers NF-κB, CREB, and MAPK pathways, enhancing expression of pro-inflammatory cytokines, metabolic enzymes, and stress-response proteins [28, 29].

In opposition, K⁺ channels—including Ca^2^⁺-activated K⁺ channels (BK, SK), voltage-gated K⁺ channels (Kv), and mechanosensitive two-pore K⁺ channels (TREK, TRAAK)—act to stabilize membrane potential: 1) Hyperpolarization through K⁺ efflux reduces Ca^2^⁺ entry and dampens excitatory signaling [30]; 2) K⁺ channels limit overactivation of inflammatory and metabolic pathways, acting as a molecular brake on TRP-mediated excitatory cascades [31].

Here we summarize the Ionic balance and Cold–Heat differentiations: 1) Ca^2^⁺-dominant signaling (TRPV + VGCC activation) → membrane depolarization, elevated metabolic flux, vasodilation, neurogenic inflammation → Heat/Yang phenotype; 2) K⁺-dominant signaling (enhanced K⁺ channel activity) → membrane stabilization, reduced Ca^2^⁺ influx, hypometabolism, vasoconstriction → Cold/Yin phenotype [26, 27].

**Fig. 3:**
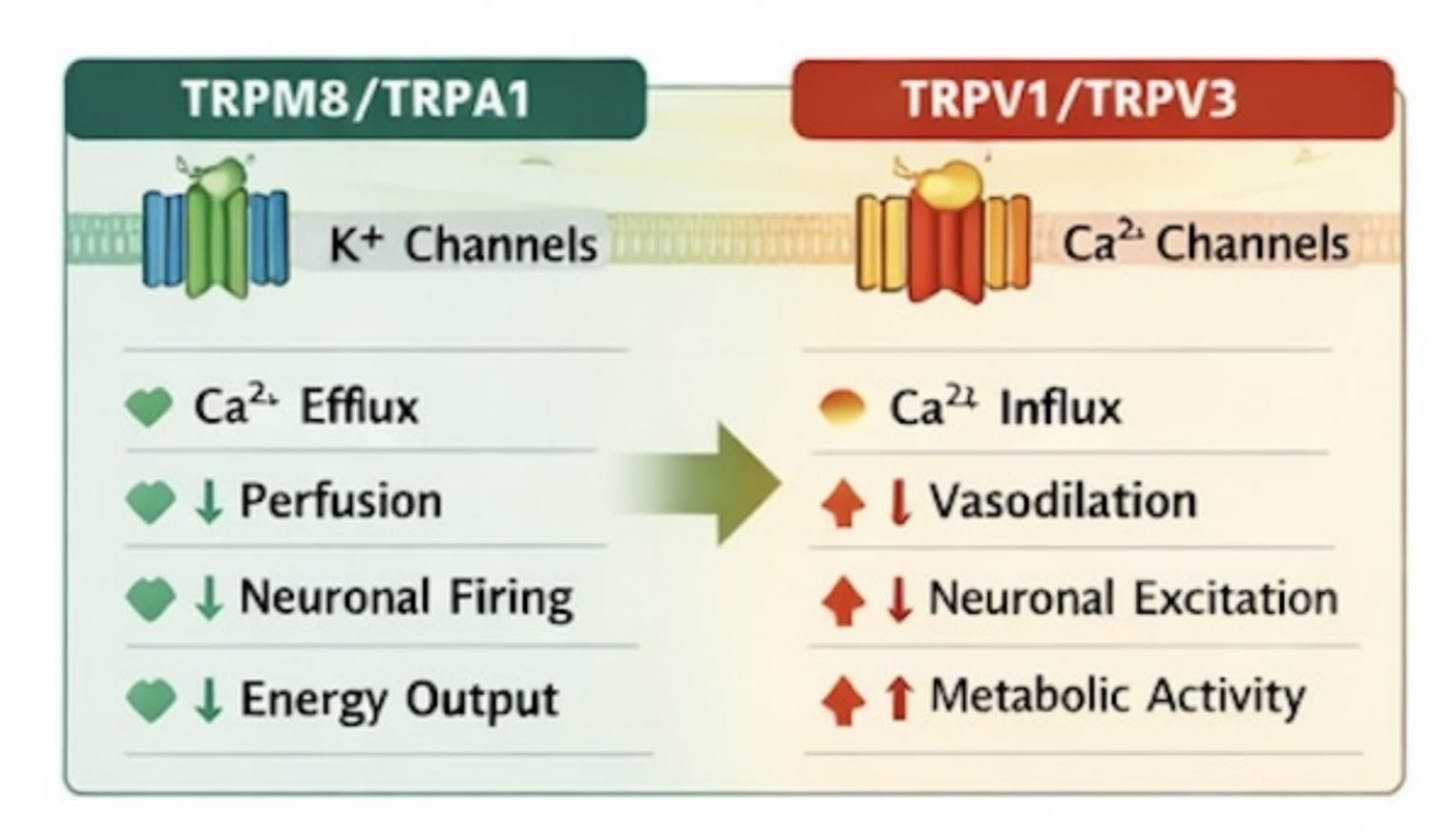
TRP, ion channels, and physiological pathways

Thus, the dynamic interplay between Ca^2^⁺ and K⁺ systems provides a molecular substrate for systemic Cold–Heat physiology, linking environmental thermosensation to vascular, metabolic, and neuroimmune outcomes [32]. The contrasting roles of Ca^2^⁺-dominant and K⁺-dominant signaling in shaping *Yin–Yang* physiological states are summarized in Table 4 [33]. Here we summarize the ionic balance and Cold–Heat differentiations in Fig. 3.

**Table 4:** Ca^2^⁺ vs. K⁺ ion channel systems in yin–yang balance

Parameter	Ca^2^⁺-Dominant Signaling	K⁺-Dominant Signaling
Membrane potential	Depolarization	Hyperpolarization
Cellular excitability	Increased	Suppressed
TRP coupling	Signal amplification	Signal stabilization
Vascular outcome	Vasodilation	Vasoconstriction
Metabolic effect	Activation	Suppression
*Yin–Yang* bias	*Yang*/Heat	*Yin*/Cold

## Cytokine executors of inflammatory balance

Cytokines translate ion-channel-mediated signaling into whole-body inflammatory and metabolic phenotypes, functioning as molecular executors of Cold–Heat differentiation [34, 35]. Representative cytokine profiles associated with *Yin*/Cold and *Yang*/Heat inflammatory states are summarized in Table 5.

**Table 5:** Cytokine profiles in cold–heat and Yin–Yang States

Cytokine	Primary function	*Yin–Yang* association
IL-1β	Fever, acute inflammation	Heat/*Yang*
IL-6	Acute-phase response	Heat/*Yang*
TNF-α	Cytotoxic inflammation	Heat/*Yang*
IL-10	Immune suppression	Cold/*Yin*
TGF-β	Immune tolerance, repair	Cold/*Yin*

*Heat/Yang patterns* involve TRPV-driven Ca^2^⁺ influx and downstream activation of NF-κB, MAPK, and inflammasome pathways increase pro-inflammatory cytokines, including IL-1β, IL-6, TNF-α, and IL-8. These cytokines mediate classical Heat/*Yang* features: fever, vasodilation, redness, vascular permeability, and heightened pain sensitivity (hyperalgesia). Positive feedback loops between cytokines and TRP/Ca^2^⁺ signaling amplify inflammatory tone, supporting sustained hypermetabolic states [11–13].

*Cold/Yin patterns involve* TRPM8/TRPA1 activation, coupled with enhanced K⁺ efflux and reduced Ca^2^⁺ entry, shifts the cytokine milieu toward anti-inflammatory dominance, with IL-10, TGF-β, and a Th2-biased profile [17, 19]. These cytokines promote immune quiescence, vasoconstriction, reduced metabolic rate, and tissue preservation, consistent with *Yin*/Cold physiological states. Cytokine-mediated signaling integrates with neural and endocrine systems to maintain systemic hyporesponsiveness to thermal or inflammatory stressors [36, 37]. Key immune cell subsets contributing to *Yin–Yang*–associated inflammatory balance are summarized in Table 6.

**Table 6:** Immune cell bias in inflammatory balance

Immune cell type	Dominant activity	*Yin–Yang* association
Th1 cells	Cellular immunity	*Yang*/Heat
Th17 cells	Auto-inflammation	*Yang*/Heat
Th2 cells	Humoral immunity	*Yin*/Cold
Regulatory T cells (Treg)	Immune tolerance	*Yin*/Cold
M1 macrophages	Pro-inflammatory	*Yang*/Heat
M2 macrophages	Tissue repair	*Yin*/Cold

The **Ca**^**2**^**⁺/K⁺ ion balance** sets the stage for cytokine output, which in turn drives **vascular, metabolic, and immune phenotypes** characteristic of Cold–Heat syndromes. This framework provides a **molecular and network-level explanation** for TCM observations, bridging TRP channel thermosensation, ionic regulation, and cytokine-mediated systemic responses [38, 39].

## Paracentral dogma: Epigenetic and glycobiological regulation of cold–heat states

The paracentral dogma extends the central dogma by incorporating dynamic, reversible regulation of molecular networks through epigenetic, transcriptomic, and post-translational modifications [40–43]. These mechanisms encode environmental, metabolic, and developmental inputs into stable yet adaptable Cold–Heat phenotypes.

### DNA methylation: Long-term thermo-inflammatory setpoints

DNA methylation at CpG islands within promoters/enhancers modulates gene transcription of TRP channels, ion channels, and cytokines [44]. Hypermethylation of IL1B, IL6, and TNF suppresses basal inflammatory tone → Cold/*Yin*-like immune phenotype. Whereas hypomethylation facilitates rapid cytokine induction → Heat/*Yang* hyperinflammatory phenotype. Meanwhile methylation of TRPV1 or TRPM8 regulatory regions adjusts sensory thresholds and excitability. In addition, Environmental influence: Chronic cold exposure, diet, stress, or early-life inflammation remodel methylation, establishing constitutional Cold–Heat bias without altering DNA sequence [43, 45].

### Histone modifications: Dynamic control of excitability and inflammation

Post-translational modifications (acetylation, methylation, glycosylations, phosphorylation) alter chromatin accessibility. H3K27ac or H3K9ac at TRPV1, CACNA1C, NFKB1 loci enhances transcriptional responsiveness → increased Ca^2^⁺ influx, neuronal excitability, cytokine release. In contrast, repressive marks (H3K9me3, H3K27me3) compact chromatin at inflammatory gene promoters → reduced excitability and anti-inflammatory bias. In this process, enzymatic regulators such as HATs, HDACs, and histone methyltransferases serve as **molecular rheostats**, linking metabolic status and environmental cues to systemic Cold–Heat balance [43, 46].

### Non-Coding RNAs: Fine-Tuning ion channels and cytokine networks

miRNAs (e.g., miR-155, miR-21, miR-146a) modulate NF-κB signaling and cytokine production, biasing responses toward Heat/*Yang* or Cold/*Yin*. From the perspective of Ion channel regulation, miRNA-mediated repression of TRPV1, CACNA1C, or KCNA3 reduces excitatory signaling → *Yin* stabilization. Reduced miRNA repression enhances *Yang*-like amplification.

In addition, lncRNAs participate in chromatin remodeling, transcriptional scaffolding, and spatiotemporal regulation of Cold–Heat gene networks, which enables rapid adaptation to environmental changes while maintaining overall systemic equilibrium [47, 48].

### Glycobiology: Post-Translational encoding of yin–yang information

N- and O-linked glycans on proteins encode metabolic and environmental information. For example, via **TRP channels,** glycosylation of TRPV1/TRPM8 modulates trafficking, gating, and ligand sensitivity → alters thermal perception and inflammatory responses. Meanwhile, **Cytokine receptors, such as** glycoforms of IL6R, TNFRSF1A, TGFBR1 regulate receptor stability, ligand affinity, and signal intensity [36, 40]. Pro-inflammatory glycoforms (e.g., agalactosylated) are involved in Heat/Yang enhancement, while anti-inflammatory glycoforms (e.g., sialylated/galactosylated) contribute to Cold/*Yin* suppression. [41–43].

In addition, in the process of IgG glycosylation, agalactosylated IgG correlates with Heat/*Yang* inflammation; sialylated IgG supports immune tolerance and Cold/*Yin* balance, showing that glycosylation provides a dynamic interface between genotype, environment, metabolism, and immune tone [41, 45, 49, 50].

Integrated Paracentral Regulation and *Yin–Yang* Stability.

Through multilayered control, DNA methylation sets long-term bias; histone modifications regulate transcriptional plasticity; non-coding RNAs allow rapid post-transcriptional tuning; glycobiology encodes metabolic and environmental context [43].

**Fig. 4:**
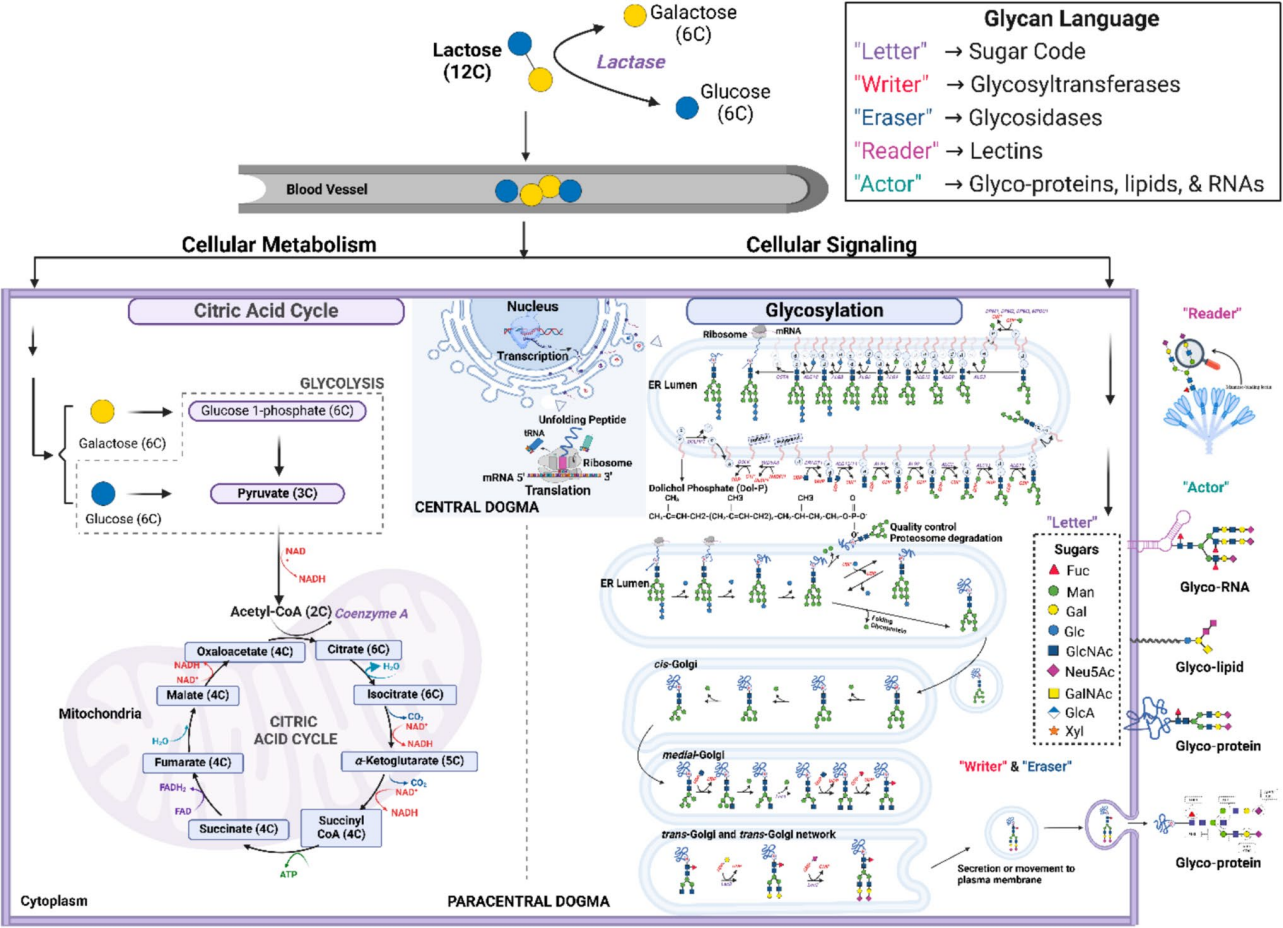
Orthodox vs paradox: Paracental dogma-supporting the central dogma with sugar code. The roles of sugars in the paths of cellular metabolism and signaling (modified from Cao et al., [43] with permission)

**Fig. 5:**
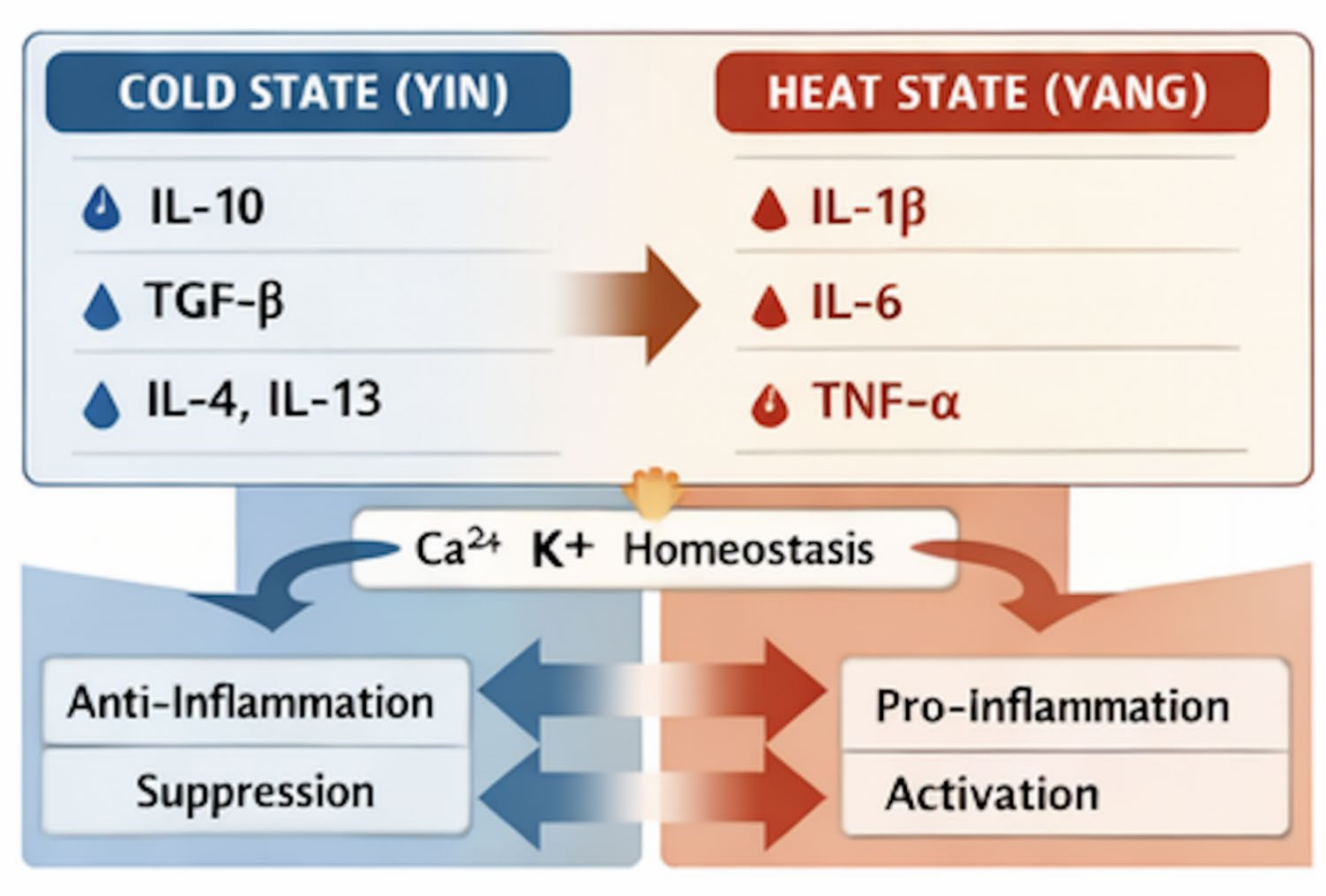
Central Dogma versus Paracentral Dogma in *Yin–Yang* regulation. Genetic encoding establishes baseline TRP and cytokine architecture, while epigenetic and glycosylation layers dynamically tune channel sensitivity, receptor signaling, and inflammatory tone

For example, at **temporal scales,** Long-term epigenetic marks, intermediate transcriptional adjustments, and rapid glycan-mediated post-translational modulation collectively stabilize Cold–Heat phenotypes while permitting reversibility. Through **system-level implication,** persistent yet modifiable Cold–Heat syndromes reflect a **biologically grounded Yin–Yang dynamic**, integrating molecular, cellular, and systemic regulation [13, 40, 46, 50]. As shown in Fig. 4 and Fig. 5, understanding paracentral dogma layers enables targeted interventions—epigenetic, transcriptomic, or glycomic—tailored to individual Cold–Heat predispositions, aligning TCM principles with precision medicine with **Translational potential** [41–43, 45, 51]. An integrated model linking TRP channels, ion homeostasis, cytokine output, and immune tone across Cold–Heat states is summarized in Table 7.

**Table 7:** Integrated inflammation balance model

System level	Cold/*Yin* dominance	Heat/*Yang* dominance
TRP channels	TRPM8, TRPA1	TRPV1–3
Ion channels	K⁺-dominant	Ca^2^⁺-dominant
Cytokines	IL-10, TGF-β	IL-1β, IL-6, TNF-α
Immune tone	Tolerance, repair	Activation, defense
Clinical pattern	Cold syndrome	Heat syndrome

## Herbal modulation and biotechnological implications

TCM classifies herbs by their “temperature nature”—Cold, Cool, Warm, or Hot—reflecting their capacity to influence systemic Cold–Heat balance. Modern mechanistic studies suggest that these herbs exert multi-target molecular effects, modulating TRP channels, ionic flux, epigenetic regulators, cytokine networks, and glycosylation pathways [38, 39, 52].

*TRP Channel Modulation.* Heat/Warm herbs (*Yang*-promoting) include examples: Ginger (Zingiber officinale), Cinnamon (Cinnamomum cassia), Capsicum (Capsicum annuum) [53, 54]. Mechanism relies on the bioactive compounds in these class of hers such as gingerol, cinnamaldehyde, and capsaicin activate TRPV1–4 channels, promoting Ca^2^⁺ influx, neuronal excitability, and local vasodilation [55, 56]. This mechanistically aligns with Heat/*Yang* phenotypes, enhancing thermogenesis and pro-inflammatory responsiveness when needed.

Whereas Cold/Cool herbs (*Yin*-promoting) are represented by the examples of Peppermint (Mentha haplocalyx), Coptis chinensis, Radix Scutellariae. Mechanically menthol and related terpenoids activate TRPM8 or TRPA1, favoring K⁺-dominant stabilization, reduced Ca^2^⁺ influx, vasoconstriction, and anti-inflammatory signaling, consistent with Cold/*Yin* systemic effects [13, 36, 40].

*Ionic and Metabolic Effects.* Herbal constituents often modulate Ca^2^⁺ and K⁺ flux in excitable tissues. For example, Ephedra (Ma Huang) contains ephedrine alkaloids that enhance Ca^2^⁺ signaling in smooth muscle and cardiac tissue, supporting *Yang*/Heat vascular and metabolic activity. However, Salvia miltiorrhiza (Danshen) flavonoids can activate K⁺ channels, reduce neuronal excitability, and protect against hyperinflammatory stress, promoting *Yin*/Cold balance [55, 56].

These multi-target effects illustrate how herbs act on TRP–Ca^2^⁺–K⁺ networks, harmonizing excitatory and inhibitory pathways to restore systemic equilibrium.

*Epigenetic and Transcriptomic Regulation.* Certain phytochemicals modulate DNA methylation, histone acetylation, and non-coding RNA networks, For example, Curcumin (Curcuma longa) inhibits histone acetyltransferases and NF-κB signaling, reducing pro-inflammatory gene expression → Cold/*Yin*-like effects [46]. In contrary, Resveratrol (Polygonum cuspidatum) influences SIRT1-mediated deacetylation, promoting anti-inflammatory cytokines (IL-10, TGF-β) while suppressing Heat/*Yang* cytokine responses. Ginsenosides (Panax ginseng) regulate miRNAs such as miR-21 and miR-146a, fine-tuning TRP channel expression and immune responses [47, 48]. These actions illustrate that herbal formulations can remodel the paracentral dogma, producing reversible changes in gene expression and excitability consistent with *Yin–Yang* modulation [44].

### Glycobiological effects

Herbs can influence glycosylation patterns of receptors, channels, and immunoglobulins, thereby tuning signal transduction. A good example is Astragalus membranaceus polysaccharides which enhance sialylation of IgG and cytokine receptors, promoting Cold/*Yin* anti-inflammatory tolerance. Another case is Rehmannia glutinosa glycosides that modulate TRP channel glycosylation, stabilizing membrane trafficking and thermal sensitivity [13, 40, 46].

This post-translational modulation allows herbs to encode environmental and metabolic signals into functional protein states, aligning with TCM Cold–Heat classification at the molecular level.

*Biotechnological and Therapeutic Implications*. At systems-level therapeutics, herbal formulas exemplify multi-modal interventions, coordinating TRP–Ca^2^⁺–K⁺ networks, cytokine balance, epigenetic plasticity, and glycosylation patterns. This contrasts with single-target drugs, offering a blueprint for polypharmacology in modern precision medicine. In terms of drug discovery inspiration, isolating bioactive compounds or combining herbs rationally can guide network pharmacology approaches, using computational modeling to predict systemic effects on Cold–Heat axes. From the perspective of personalized interventions: Understanding genetic, epigenetic, and glycomic backgrounds can enable tailored herb-based therapies, optimizing therapeutic outcomes based on individual Cold–Heat tendencies [57, 58].

In Summary, herbs traditionally classified by thermal nature provide mechanistic leverage points across TRP channels, ion flux, epigenetics, and glycosylation, demonstrating that TCM formulations operate as coordinated, multi-target regulatory systems. This integrated approach not only validates classical TCM concepts at a molecular level but also inspires next-generation therapeutics that balance excitatory and inhibitory pathways for systemic homeostasis.

## Expert outlook: Emerging biotechnological applications and translational opportunities from TCM and PPPM perspectives

1. TRP-Based Biosensors for Inflammatory and Thermal States

Thermosensitive TRP channels, including TRPV1, TRPA1, TRPM8, and TRPC subtypes, are increasingly recognized as versatile molecular modules for biosensing applications due to their inherent sensitivity to temperature, pH, reactive oxygen species, and lipid-derived mediators [35]. Engineered TRP-based biosensors leverage these properties by coupling ion-channel activation to measurable readouts such as changes in membrane potential, intracellular calcium flux, or optogenetically encoded fluorescent signals [34]. For instance, TRPV1-expressing cells embedded in microfluidic chips can report local tissue heating or inflammatory mediator accumulation in real time, while TRPM8-based sensors can detect subtle cooling stimuli relevant to Cold-pattern physiology in Traditional Chinese Medicine (TCM). Integration with organ-on-chip systems allows monitoring of complex multi-tissue interactions, such as immune-endothelial crosstalk under inflammatory stress [26, 27]. Wearable bioelectronic platforms incorporating TRP-based sensors could provide continuous monitoring of physiological Cold–Heat fluctuations, enabling individualized lifestyle interventions, early detection of inflammatory exacerbations, or adaptive dosing of TRP-modulating compounds, including herbal or nutraceutical agents [36, 37].

Future directions include the development of multiplexed TRP arrays capable of simultaneous detection of diverse thermal, chemical, and metabolic inputs, combined with real-time AI-assisted analytics to quantify Cold–Heat phenotypes at single-cell or tissue levels.

2) Epigenetic and Glycan Biomarkers of Cold–Heat States

Epigenetic modifications and protein glycosylation patterns provide robust, dynamic biomarkers for system-level Cold–Heat status, bridging molecular signaling with physiological phenotype. DNA methylation at promoter regions of TRP channels (e.g., TRPV1, TRPM8) or cytokine genes, histone acetylation at NF-κB–responsive loci, and regulatory microRNAs collectively modulate ion-channel expression and inflammatory responsiveness. These molecular signatures reflect both chronic predispositions (endogenous Yin–Yang imbalances) and acute responses to environmental stimuli [59].

3) Glycobiology adds a complementary post-translational layer

For example, differential N-linked sialylation or fucosylation of TRP channels or cytokine receptors can alter channel gating, membrane trafficking, and ligand sensitivity, thus influencing Cold–Heat physiology. High-mannose structures on immune receptors may correlate with heightened inflammatory responsiveness in Heat-pattern states, while hypo-sialylation may mark Cold-pattern susceptibility [43]. Recent advances in mass spectrometry-based glycomics, lectin microarrays, and single-cell multi-omics now enable detailed profiling of these glyco-epigenetic signatures from blood, saliva, or tissue biopsies [41, 42, 49, 50].

4) Translationally, these biomarkers can support: a. Objective stratification of patients into Cold–Heat phenotypes for PPPM; b. Monitoring of response to TRP-targeted interventions, herbal therapies, or dietary modulation; 3) Identification of individualized glyco-epigenetic targets for novel biopharmaceuticals [55].

5) AI-Driven Cold–Heat Stratification and Systems Modeling for TCM and PPPM

The Cold–Heat framework in TCM can be conceptualized as an emergent property of multi-layered biological networks encompassing ion channels, immune signaling, metabolism, and epigenetic regulation. Artificial intelligence (AI) and machine learning provide the computational tools to integrate heterogeneous datasets and uncover latent Cold–Heat patterns [52]. By combining TRP and ion-channel expression/activity, Cytokine and metabolite profiles, Epigenetic modifications and microRNA networks, Glycosylation signatures, Physiological readouts (body temperature, heart rate variability, skin conductance), and Patient-reported TCM symptoms*,* AI models can identify non-linear interactions and predictive signatures that may elude conventional statistical analysis [55, 56]. For example, deep learning frameworks could predict shifts from Cold- to Heat-pattern inflammatory states, estimate susceptibility to thermal stress, or suggest personalized interventions (pharmacological or lifestyle-based) targeting specific TRP–ion channel circuits [60].

**Fig. 6:**
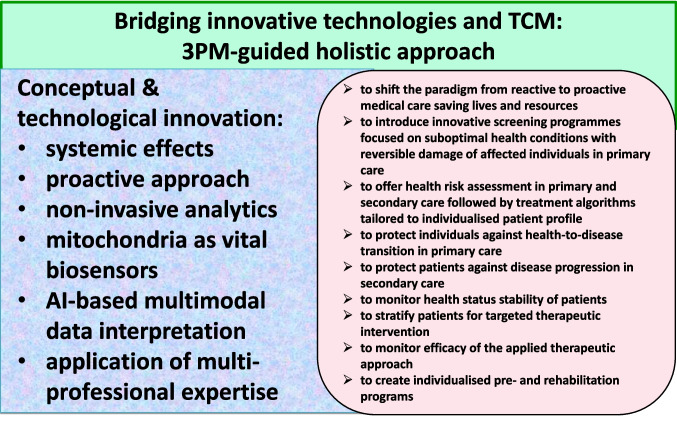
3PM-guided holistic approach is bridging advances of TCM with innovative technologies to advance healthcare saving lives and resources

Beyond clinical stratification, AI-enabled Cold–Heat modeling offers a blueprint for rational design of bioinspired therapeutics. Synthetic biology approaches could engineer TRP-modulating constructs, glycoengineered cytokines, or epigenetic modulators guided by AI-derived network insights. These strategies collectively position Cold–Heat biology at the interface of TCM, molecular physiology and systems pharmacology, opening avenues for PPPM-driven healthcare [13, 46]. Figure 6 summarizes the proposed conceptual and technological innovation of the 3PM-guided approach.

## Data Availability

No datasets were generated, nor analyzed during the current study.

## Abbreviations

BC: Breast Cancer
CACNA1C: Voltage-dependent L-type Calcium Channel Subunit Alpha-1C
CGRP: Calcitonin Gene-related Peptide
FSP: Flammer Syndrome Phenotype
H3K27ac: Acetylation of Lysine 27 on Histone H3
H3K9ac: Acetylation of Histone H3 at Lysine 9
IL: Interleukins
lncRNA: Long Non-Coding Ribonuclear Acids
miRNA: Micro Ribonuclear Acids
NFKB1: Nuclear Factor Kappa B Subunit 1
NK cells: Natural Killer Cells
PPPM/3PM: Predictive, Preventive and Personalized Medicine
TCM: Traditional Chinese Medicine
TGF: Tumor Necrosis Factors
Treg: Regulatory T Cells
TRP: Transient Receptor Potential
TRPV: Transient Receptor Potential Voltage
VGCCs: Voltage-gated Ca^2^⁺ Channels
WHO: World Health Organization
